# Morpho-molecular approach reveals three novel endophytic fungi in *Polyschema* (Pleosporales, Latoruaceae) associated with roots of baobab trees in Yunnan, China

**DOI:** 10.3897/mycokeys.129.182259

**Published:** 2026-03-09

**Authors:** Fangqi Sun, Hongbo Jiang, Jaturong Kumla, Rungtiwa Phookamsak, Junfu Li, Yunju Li, Jianchu Xu, Jayarama Darbhe Bhat, Nakarin Suwannarach

**Affiliations:** 1 Department of Biology, Faculty of Science, Chiang Mai University, Chiang Mai 50200, Thailand Yunnan Key Laboratory for Wild Plant Resources, Kunming Institute of Botany, Chinese Academy of Sciences Kunming China; 2 Center of Excellence in Microbial Diversity and Sustainable Utilization, Chiang Mai University, Chiang Mai 50200, Thailand Centre for Mountain Futures (CMF), Kunming Institute of Botany Kunming China; 3 Key Laboratory of Economic Plants and Biotechnology, Yunnan Key Laboratory for Wild Plant Resources, Kunming Institute of Botany, Chinese Academy of Sciences, Kunming 650201, Yunnan, China College of Science, King Saud University Riyadh Saudi Arabia; 4 Honghe Center for Mountain Futures, Kunming Institute of Botany, Chinese Academy of Sciences, Honghe County 654400, Yunnan, China Faculty of Science, Chiang Mai University Chiang Mai Thailand; 5 Centre for Mountain Futures (CMF), Kunming Institute of Botany, Kunming 650201, Yunnan, China Center of Excellence in Microbial Diversity and Sustainable Utilization, Chiang Mai University Chiang Mai Thailand; 6 CIFOR-ICRAF China Program, World Agroforestry (ICRAF), Kunming 650201, Yunnan Province, China Honghe Center for Mountain Futures, Kunming Institute of Botany, Chinese Academy of Sciences Honghe County China; 7 Department of Botany and Microbiology, College of Science, King Saud University, P.O. Box 22452, Riyadh 11495, Saudi Arabia CIFOR-ICRAF China Program, World Agroforestry (ICRAF) Kunming China; 8 Vishnugupta Vishwavidyapeetam, Ashoke, Gokarna 581326, India Vishnugupta Vishwavidyapeetam Gokarna India

**Keywords:** *Adansonia digitata*, dark septate endophytes, Dothideomycetes, new fungal species, phylogeny, taxonomy

## Abstract

Endophytic fungi associated with roots of the baobab tree (*Adansonia digitata* L.) remain poorly documented in terms of species diversity worldwide. During an ongoing fungal survey in the dry-hot valleys of Honghe (Yunnan Province, China), five endophytic fungal strains were isolated from healthy roots of *A. digitata*. Based on multi-locus phylogenetic analyses of four loci (ITS, LSU, SSU, and *rpb*2), combined with morphological observations, all five strains were identified as belonging to the genus *Polyschema*. Hence, three novel species are introduced in the present study, namely *P. adansoniae-digitatae*, *P. hongheense*, and *P. radicicola*. This study constitutes the first record of *Polyschema* from mainland China. Detailed morphological descriptions, illustrations, and phylogenetic placements of the three new species are provided. Additionally, the species diversity, morphology, and phylogeny of *Polyschema* are updated and discussed. These findings enrich knowledge of endophytic fungal diversity associated with baobab in China and provide insights into the taxonomy and diversity of *Polyschema*. Furthermore, an updated discussion on the ecology and genera within the family Latoruaceae is presented.

## Introduction

Latoruaceae is a family within Pleosporales, Dothideomycetes, that was introduced by Crous et al. (2015a) to accommodate the genera *Latorua* and *Polyschema*, with *Latorua* designated as the type genus and *Latorua caligans* as the type species. The teleomorph of Latoruaceae is characterized by immersed, compressed, globose ascomata with a papillate ostiole, 8-spored, bitunicate, fissitunicate, cylindrical to clavate asci with a clear ocular chamber, and 1–3-septate, fusiform, brown ascospores (Ariyawansa et al. 2015). The teleomorph is mostly presented as dark brown, verruculose hyphae (Crous et al. 2024) and is characterized by erect, moniliform, brown conidiophores or reduced conidiogenous cells and smooth to verruculose, brown, polyblastic conidiogenous cells or reduced inconspicuous loci on hyphae, bearing smooth or verrucose, septate, fusoid-ellipsoidal conidia in chains or solitary, sometimes becoming cupulate, with secondary conidia and cells or septa darker pigmented than the rest of the conidium (Crous et al. 2015a). Besides *Latorua*, six other genera, including *Matsushimamyces* (Sharma et al. 2015), *Multiverruca* (Wang et al. 2023), *Polyschema* (Crous et al. 2015a), *Pseudoasteromassaria* (Ariyawansa et al. 2015), *Triseptata* (Boonmee et al. 2020), and *Verrucohypha* (Crous et al. 2024), have been accommodated within the family. Genera within Latoruaceae are mainly distributed in North America (Ellis 1976; Shearer 1982), South America (Crous et al. 2015a; Crous et al. 2024), Africa (Crous et al. 2015a), East Asia (Ariyawansa et al. 2015; Wang et al. 2023), South Asia (Ellis 1976; Boonmee et al. 2020), and Australia (Matsushima 1989; Tan and Bishop-Hurley 2024). They are predominantly saprobic, associated with plant leaves (Ariyawansa et al. 2015), roots (Crous et al. 2024; Crous et al. 2025), soil (Wang et al. 2023), and decaying wood (Mota et al. 2008). Furthermore, some species in *Verrucohypha* and *Polyschema* are reported to be endophytic and associated with roots (Crous et al. 2024; Crous et al. 2025), while *Polyschema sclerotigenum* and *Pseudoasteromassaria fagi* were reported as a human pathogenic fungi (Crous et al. 2015b) and a plant pathogenic fungus, respectively (Ariyawansa et al. 2015).

Several genera within Latoruaceae are considered monotypic. The genus *Latorua* became monotypic following the recombination of *L. grootfonteinensis* into *Bahusandhika* (Crane and Miller 2016). *Pseudoasteromassaria* was incorporated into Latoruaceae by Ariyawansa et al. (2015) to accommodate the type species, *Ps. fagi*, associated with the twigs of *Fagus crenata* (Fagaceae) in Japan, based on morphological observations and combined SSU and LSU phylogenetic analyses. Subsequently, two freshwater species from Thailand, *Ps. aquatica* (Dong et al. 2020) and *Ps. spadicea* (Tibpromma et al. 2017), were accommodated in this genus. *Matsushimamyces* was established to accommodate keratinophilic fungi from soil in central India. *Matsushimamyces bohaniensis* (the type species) and *M. venustus* (≡ *Polyschema venustum*) were isolated from decaying leaves of an unidentified tree in Cuba (Sharma et al. 2015). *Multiverruca* was introduced by Wang et al. (2023) as a monotypic genus to accommodate a thermotolerant fungus isolated from soil in Zhejiang, China. Based on a combined LSU, ITS, and SSU phylogenetic analysis, *Triseptata* was introduced to accommodate a single species, *T. sexualis*, isolated from dead branches of an unidentified plant in Thailand (Boonmee et al. 2020). Later, Tan and Bishop-Hurley (2024) included *T. podargi-strigoidis* [as “podargusstrigoides”], isolated from decayed wood of *Acacia* in Queensland, Australia. *Verrucohypha* was assigned to Latoruaceae based on an ITS phylogenetic analysis, forming a clade closely related to *Polyschema*, and is the most recently described genus in this family (Crous et al. 2024). The genus was introduced as monotypic to accommodate the dark septate endophyte, *V. endophytica* (type species), isolated from roots of *Acrocomia aculeata* (Arecaceae) in Brazil (Crous et al. 2024).

*Polyschema* is a species-rich genus in Latoruaceae compared to other genera in this family. The genus was introduced by Upadhyay (1966) with *P. terricola* as its type species. The teleomorph of species within *Polyschema* remains undiscovered. However, the hyphomycetous asexual morphological characteristics were described as micronematous or semi-macronematous, mononematous conidiophores, mono- or polytretic, smooth or echinulate, determinate, discrete or integrated conidiogenous cells, bearing ellipsoidal, cylindrical, clavate, or obclavate, septate, pigmented, smooth to verrucose or echinulate conidia (Ellis 1976; Hongsanan et al. 2020). Species of *Polyschema* lack comprehensive modern taxonomic treatment. Based primarily on morphological features, species within the *Polyschema* were divided into two distinct groups on the basis of their conidial surfaces by Rao and Reddy (1984), and with further refinement by Mota et al. (2008). In accordance with Rao and Reddy (1984) and Mota et al. (2008), the first group (group I) comprises taxa with verrucose or echinulate conidia, including *P. chambalense* (Joshi et al. 1983), *P. congolense* (Reisinger and Kiffer 1974), *P. indicum*, *P. larviforme*, *P. olivaceum* (Ellis 1976), *P. queenslandicum* (Matsushima 1989), *P. sagari* (Pandeya and Saksena 1978), *P. terricola* (type species; Ellis 1976), *P. variabile* (Tiwari et al. 1977), and *P. yakuense* (Matsushima 1975). The second group (group II) comprises taxa with smooth-walled conidia, including *P. bicellulare* (Shearer 1982), *P. clavulatum* (Ellis 1976), *P. cubense* (Mercado-Sierra and Mena-Portales 1992), *P. lignicola* (Rao and Reddy 1984), *P. nigroseptatum* (Mota et al. 2008), and *P. obclaviforme* (Ruiz et al. 2000). *Polyschema venustum* was reassigned to the genus *Matsushimamyces* according to a combined morphological and ITS-LSU phylogenetic analysis (Sharma et al. 2015).

Previously, the phylogenetic position and relationships of *Polyschema* within Pleosporales remained unclear. Most species placed in this genus were based on morphological characteristics and were introduced in the 19^th^ century. There are 22 species accepted as members of *Polyschema* (Index Fungorum 2025; accessed 04 November 2025). Furthermore, 18 species of *Polyschema* lack molecular data to clarify their phylogenetic placement. Shenoy et al. (2010) revealed the preliminary phylogenetic affinity of *Polyschema* in Pleosporales based on phylogenetic analyses of LSU sequence data, which placed the genus as a monophyletic lineage related to *Lentithecium* and *Leptosphaeria* in Pleosporales. Presently, molecular data are available for only five species of *Polyschema*, viz. *P. congolense*, *P. larviforme*, *P. sclerotigenum*, *P. terricola* (Hongsanan et al. 2020), and *P. endophytica* (Crous et al. 2025). Additionally, most available phylogenetic markers in the above five taxa used to identify species within *Polyschema* include only ITS and LSU data.

Species within *Polyschema* typically appear saprobic in various habitats, including forest soil (Crous et al. 2015b), grassland soil, decaying wood (Ellis 1976), leaves (Ruiz et al. 2000), and old firewood (Ellis 1976). In rare cases, they have been found to be pathogenic in human clinical tissue (Crous et al. 2015b) or endophytic in healthy roots of coffee trees (Crous et al. 2025). These species are mainly distributed across North America (Ellis 1976; Shearer 1982; Mota et al. 2008; Crous et al. 2015b), South America (Ellis 1976; Mercado-Sierra and Mena-Portales 1992; Ruiz et al. 2000; Castañeda Ruíz et al. 2009; Crous et al. 2025), East Asia (Matsushima 1975, 1980), and South Asia (Ellis 1976; Tiwari et al. 1977; Pandeya and Saksena 1978; Joshi et al. 1983). As individual cases, *P. queenslandicum* has been reported exclusively from Australia (Matsushima 1989), and *P. congolense* from Africa (Ellis 1976).

While conducting fieldwork to collect endophytic fungi associated with higher plants in Yunnan Province, China, five *Polyschema* strains were isolated from healthy roots of the baobab tree (*Adansonia digitata*). Through morphological comparisons coupled with multi-locus phylogenetic analyses based on a combined dataset of ITS, LSU, SSU, and *rpb*2, three novel species in *Polyschema* (Latoruaceae) from Yunnan are introduced and illustrated in the present study. Additionally, the ecological information and genera of the family Latoruaceae are updated.

## Materials and methods

### Sample collection, fungal isolation, examination, and preservation

Fresh, healthy roots of baobab trees from Honghe County (altitude 443.2–510.3 m), Honghe Prefecture, Yunnan Province, China, were collected on 25 June 2024. Samples were enclosed in sealed plastic Ziploc bags, stored in cooling boxes, and transported to the laboratory within 24 hours, accompanied by labels containing collection details. Endophytes were isolated from root samples following the protocols outlined by Mattoo and Nonzom (2022), Suwannarach et al. (2023), and Luo et al. (2024). Each sample was cut into 1 × 1 cm^2^ pieces after being thoroughly cleaned with running tap water. Root fragments were surface sterilized using 75% ethanol for 4 min, 5% sodium hypochlorite (NaOCl) for 3 min, and 80% ethanol for 30 s, followed by three rinses with sterilized water and drying with sterile paper tissues. Root pieces were then transferred onto potato dextrose agar (PDA) plates with 0.03% rose Bengal and incubated at 25–30 °C in the dark for 2–5 days. Mycelia emerging from the cut end of root pieces were picked using sterilized needles, purified, and subcultured onto new PDA plates when individual hyphal tips grew out from the root pieces. After purification, the cultures were incubated at 25–30 °C for 1–2 months under dark conditions for sporulation and morphological examination (Mattoo and Nonzom 2022). Culture characteristics, growth, and sporulation on the medium were observed and recorded. Pure fungal cultures are preserved on PDA slants for short-term storage and in 20% glycerol at −20 °C for long-term preservation. Dry culture materials were deposited in the herbarium of the Cryptogams, Kunming Institute of Botany, Academia Sinica (KUN–HKAS). Living cultures were conserved at the Kunming Institute of Botany Culture Collection (KUNCC). New fungal taxa were registered in the MycoBank database (MycoBank 2025).

### Microscopic examination

Micromorphological features were examined and captured using a Nikon ECLIPSE Ni-U compound microscope equipped with a Nikon DS-Ri2 camera. All morphological characteristics (e.g., conidiophore, conidiogenous cell, conidia, and chlamydospores) were measured using the Tarosoft® Image Framework version 0.9.7. Photographic plates were edited and combined in Adobe Photoshop CS6 (Adobe Systems Inc., United States) to present the size and morphology of the above-mentioned micromorphological features.

### DNA extraction, PCR amplification, and sequencing

Fungal genomic DNA was extracted from fresh mycelia grown on PDA for 5 days at 25 °C using the Biospin Fungus Genomic DNA Extraction Kit (BioFlux®, Hangzhou, China) according to the manufacturer’s instructions. The internal transcribed spacer (ITS: ITS1-5.8S-ITS2), 28S large subunit rDNA (LSU), 18S small subunit rDNA (SSU), and RNA polymerase II second largest subunit (*rpb*2) loci were amplified using the primer pairs ITS5/ITS4 (White et al. 1990), LR0R/LR5 (Vilgalys and Hester 1990), NS1/NS4 (White et al. 1990), and fRPB2-5F/fRPB2-7cR (Liu et al. 1999), respectively. The PCR reaction mixture was performed in a total volume of 25 µL, containing 2 µL of DNA template, 1 µL of each forward and reverse primer (10 µM), 12.5 µL of 2 × Power Taq PCR Master Mix (mixture of EasyTaq™ DNA Polymerase, dNTPs, and optimized buffer; Beijing Bio Teke Corporation, China), and 8.5 µL of double-distilled water (ddH_2_O). The thermal cycling conditions for PCR amplification of ITS, LSU, SSU, and *rpb*2 followed those described by Xu et al. (2022). PCR products were sent to TsingKe Biological Technology (Beijing) Co., Ltd., China, for purification and sequencing. All newly generated consensus sequences were deposited in the GenBank database, and the corresponding accession numbers are presented in Table 1.

**Table 1.** Taxa used in this study, along with their corresponding GenBank accession numbers. Ex-type strains are indicated with the superscript “T.” The taxa obtained in this study are in bold. “–” indicates the absence of sequence data in GenBank.

Species names	Strain numbers	GenBank accession numbers			
		ITS	LSU	SSU	*rpb*2
*Bahusandhika grootfonteinensis*	CBS 369.72	–	KR873267	–	–
*Bahusandhika indica*	MTCC 11761	KF460273	KF460274	–	–
*Bahusandhika indica*	MFLUCC 23-0304	PP809083	PP800322	PP811805	–
*Bahusandhika* sp.	MFLU FT1688	PV870593	PV992408	PV996961	–
*Falciformispora senegalensis*	CBS 196.79^T^	KF015673	KF015631	KF015636	KF015717
*Falciformispora tompkinsii*	CBS 200.79^T^	NR132041	KF015625	KF015639	KF015719
*Latorua caligans*	CBS 576.65^T^	KR873232	KR873266	–	–
*Lentimurispora urniformis*	MFLUCC 18-0497	–	MH179144	MH179160	–
*Matsushimamyces bohaniensis*	CBEC001^T^	KP765516	KR350633	–	–
*Matsushimamyces venustus*	CBS:140212^T^	KT428157	KT428158	–	–
*Multiverruca sinensis*	CGMCC 3.20956^T^	ON230024	ON230021	–	–
*Multiverruca sinensis*	GZUIFR 22.040	ON230025	ON230022	–	–
*Multiverruca sinensis*	GZUIFR 22.041	ON230026	ON230023	–	–
***Polyschema adansoniae-digitatae***	**KUNCC25-20156 ^T^**	**PX632669**	**PX632690**	**PX632708**	**PX531817**
***Polyschema adansoniae-digitatae***	**KUNCC25-20157**	**PX632670**	**PX632691**	**PX632709**	**PX531818**
*Polyschema congolense*	CBS:542.73^T^	MH860770	MH872486	–	EF204486
*Polyschema endophytica*	COAD3972^T^	PV206769	PV206770	–	–
***Polyschema hongheense***	**KUNCC25-20158 ^T^**	**PX632671**	**PX632692**	**PX632710**	**PX531819**
*Polyschema larviforme*	CBS 463.88^T^	–	EF204503	–	–
*Polyschema larviforme*	ILLS00171087	MH472659	–	–	–
***Polyschema radicicola***	**KUNCC25-20159 ^T^**	**PX632672**	**PX632693**	**PX632711**	**PX531820**
***Polyschema radicicola***	**KUNCC25-20160**	**PX632673**	**PX632694**	**PX632712**	**PX531821**
*Polyschema sclerotigenum*	UTHSC DI14-305^T^	NR137973	KP769976	–	–
*Polyschema sclerotigenum*	RMS	MF139128	–	–	–
*Polyschema terricola*	CBS 301.65^T^	MH858576	MH870213	NG061058	EF204487
*Pseudoasteromassaria aquatica*	MFLUCC 18-1397^T^	MT627674	MN913721	MT864322	–
*Pseudoasteromassaria fagi*	HHUF 30471^T^	NR154376	NG_059805	NG061265	–
*Pseudoasteromassaria fagi*	HHUF 30472	LC061595	LC061590	–	–
*Pseudoasteromassaria spadicea*	MFLUCC 15-0973^T^	KY522726	KY522724	–	–
*Triseptata podargi-strigoidis*	BRIP 76063a^T^	NR_191342	NG_243442	–	–
*Triseptata sexualis*	MFLUCC:11-0002^T^	MN977832	MN977833	MN977850	–
*Verrucohypha endophytica*	COAD 3604^T^	PP913763	PP913764	–	–

Abbreviations: CBS: Westerdijk Fungal Biodiversity Institute, Utrecht, Netherlands; CCF: Culture Collection of Fungi, Charles University in Prague; CGMCC: China General Microbiological Culture Collection Center, Beijing, China; MFLUCC: Mae Fah Luang University Culture Collection, Chiang Rai, Thailand; ILLS: Illinois Natural History Survey Fungarium, Champaign, Illinois, USA; UTHSC: University of Tennessee Health Science Center, Memphis, Tennessee, USA; RMS: Wilhelm G. Solheim Mycological Herbarium (Rocky Mountain Herbarium), University of Wyoming, Laramie, Wyoming, USA; CBEC: Center for Biodiversity Exploration and Conservation, Bhopal, Madhya Pradesh, India; GZUIFR: Institute of Fungus Resources, Guizhou University, Huaxi, Guiyang, China; HHUF: Hirosaki University Fungarium, Hirosaki, Aomori, Japan; BRIP: Queensland Plant Pathology Herbarium, Department of Agriculture and Fisheries, Dutton Park, Brisbane, Queensland, Australia; COAD: Center for Mycological Research, Guizhou University, Guiyang, Guizhou, China; KUNCC: Culture Collection of the Herbarium of Cryptogams Kunming Institute of Botany, Academia Sinica, Kunming, China.

### Sequence alignment and phylogenetic analyses

Sequence data for all loci were subjected to a BLAST search to identify sequences closely related to the five new fungal strains (KUNCC25-20156, KUNCC25-20157, KUNCC25-20158, KUNCC25-20159, and KUNCC25-20160) in the NCBI database (https://blast.ncbi.nlm.nih.gov/Blast.cgi; accessed 25 September 2025). Reference sequences from relevant publications and BLAST results of closely related species were downloaded from GenBank to supplement the datasets (Table 1). To determine the accurate phylogenetic placement of the five new strains, a concatenated ITS–LSU–SSU–*rpb*2 phylogenetic tree was analyzed using maximum likelihood (ML) and Bayesian inference (BI) methods. Initially, individual DNA sequence matrices were aligned using the online platform MAFFT v. 7.511 (Katoh et al. 2019). The resulting alignments were then trimmed and refined as necessary using BioEdit v. 6.0.7 (Hall 1999). Missing nucleotide bases at the start and end of the consensus sequences were trimmed. ML analysis was performed using the CIPRES Science Gateway v. 3.3 platform (Miller et al. 2010), employing the RAxML-HPC v.8 on XSEDE (version 8.2.12) software. Analyses utilized default parameters except for the implementation of the GAMMA nucleotide substitution model and the execution of 1,000 rapid bootstrap replicates (MLBS). Individual locus datasets were first analyzed by ML to assess the congruence of tree topologies, after which the concatenated ITS–LSU–SSU–*rpb*2 sequence matrix was analyzed.

For BI analysis, the optimal nucleotide substitution model was independently determined for each locus using MrModeltest 2.3 (Nylander 2008). Based on the Akaike Information Criterion (AIC), the GTR+I+G model was selected as the best-fitting model for ITS, LSU, SSU, and *rpb*2 loci. BI analyses were conducted via the online portal CIPRES Science Gateway v. 3.3 (Miller et al. 2010) using MrBayes v. 3.2.7 (Ronquist and Huelsenbeck 2003), with Markov chain Monte Carlo (MCMC) sampling to estimate posterior probabilities (PP). Specifically, six concurrent Markov chains were run for 2,000,000 generations, with trees sampled every 100^th^ generation. A burn-in of 25% was applied, and the analysis was automatically terminated once the mean standard deviation of split frequencies fell below 0.01, following the criteria outlined by Maharachchikumbura et al. (2015).

The tree topologies generated in this study were visualized using FigTree v. 1.4.0 (Rambaut 2016). The phylogram was edited and formatted using Microsoft Office PowerPoint 2016 (Microsoft Inc., Redmond, WA, USA) and converted to a TIFF file with Adobe Photoshop CS6 (Adobe Systems Inc., San Jose, CA, USA). The final phylogram was submitted to Figshare (doi: 10.6084/m9.figshare.30716888; https://www.figshare.com; accessed 26 November 2025).

## Results

### Phylogenetic analyses

The concatenated ITS–LSU–SSU–*rpb*2 sequence dataset comprises 26 strains of representative species within Latoruaceae and four strains of representative species within Lentimurisporaceae, with *Falciformispora senegalensis* (CBS 196.79) and *F. tompkinsii* (CBS 200.79) as the outgroup taxa. The dataset consists of 5,368 total characters, including gaps (ITS: 1–1089 bp, LSU: 1090–2851 bp, SSU: 2852–4260 bp, *rpb*2: 4261–5368 bp). The final optimized likelihood score for the ML analysis was −19,187.116587. All free model parameters were estimated by the RAxML GAMMA model of rate heterogeneity, with 1,096 distinct alignment patterns and 68.51% undetermined characters or gaps. Estimated base frequencies were as follows: A = 0.248430, C = 0.233171, G = 0.273844, T = 0.244555, with substitution rates AC = 1.489123, AG = 2.533300, AT = 1.245085, CG = 0.740529, CT = 6.110617, GT = 1.000000. The gamma distribution shape parameter alpha = 0.160265, and the tree length = 1.397439. The final average standard deviation of split frequencies at the end of the total MCMC generations was calculated as 0.008629 in the BI analysis.

**Figure 1.**
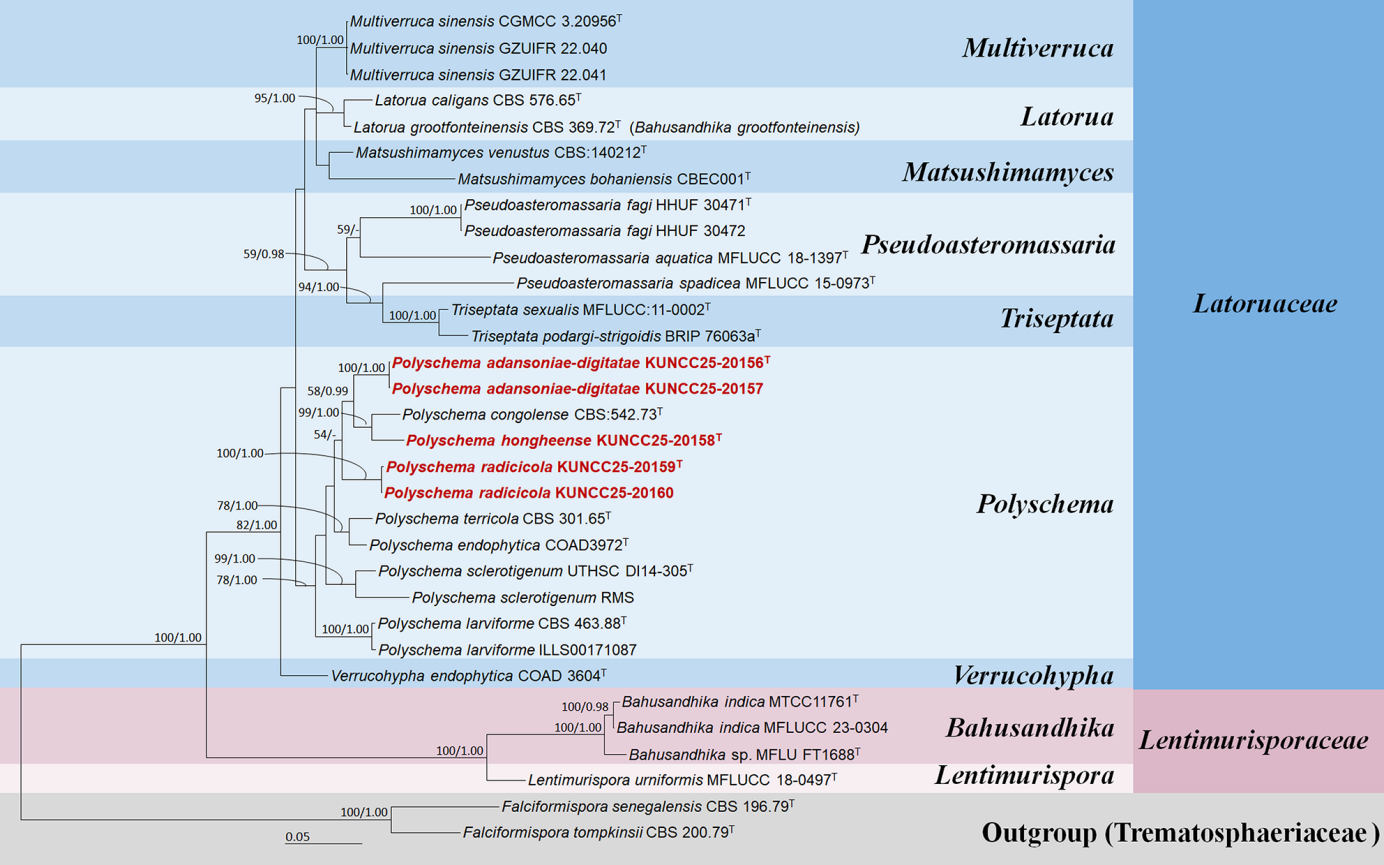
Bayesian inference tree generated by MrBayes v. 3.2.7 on XSEDE in the CIPRES Science Gateway based on a concatenated ITS, LSU, SSU, and *rpb*2 sequence dataset. The tree is rooted to *Falciformispora senegalensis* (CBS 196.79) and *F. tompkinsii* (CBS 200.79). Maximum likelihood bootstrap values (MLBS) greater than 50% and Bayesian posterior probabilities (BYPP) greater than 0.95 are written above the nodes as MLBS/BYPP. Ex-type strains are labeled with the superscript “T,” and newly generated sequences are in red bold.

Tree topologies generated based on ML and BI analyses were similar in the present study. The phylogenetic tree, constructed through analyses of the combined ITS, LSU, SSU, and *rpb*2 sequence dataset, demonstrated the relationships among taxa within Latoruaceae. Therefore, the phylogenetic tree obtained from the BI analysis was selected and is presented in Fig. 1. These findings are consistent with phylogenetic trees from previous studies (Crous et al. 2024; Crous et al. 2025). The present phylogenetic analyses revealed that seven genera, viz. *Latorua*, *Matsushimamyces*, *Multiverruca*, *Polyschema*, *Pseudoasteromassaria*, *Triseptata*, and *Verrucohypha*, were placed in Latoruaceae. In the phylogenetic tree, two new strains, KUNCC25-20156 and KUNCC25-20157, formed a robust subclade and have a close relationship with *P. congolense* (CBS 542.73) and *P. hongheense* (KUNCC 25-20158) (58% MLBS and 0.99 BYPP; Fig. 1). The new strain KUNCC25-20158 formed a separate branch and is sister to *P. congolense* (CBS 542.73) with strong statistical support (99% MLBS and 1.00 BYPP; Fig. 1). In addition, the other two new strains (KUNCC25-20159 and KUNCC25-20160) formed a robust subclade basal to *P. adansoniae-digitatae*, *P. congolense*, and *P. hongheense* with 54% MLBS support in the ML analysis but low support in the BI analysis (Fig. 1). Notably, *Pseudoasteromassaria spadicea* (MFLUCC 15-0972) did not cluster with *Ps. aquatica* (MFLUCC 18-1397) and *Ps. fagi* (HHUF 30471, HHUF 30472) in *Pseudoasteromassaria*, concurring with previous studies (Crous et al. 2024; Crous et al. 2025). The species formed a distinct branch clustering with *Triseptata sexualis* (MFLUCC 11-0002) and *T. podargi-strigoidis* (BRIP 76063a) with high statistical support (94% MLBS and 1.00 BYPP; Fig. 1) in the present study. Furthermore, *Bahusandhika grootfonteinensis* (≡ *Latorua grootfonteinensis*) formed a strongly supported subclade with *Latorua caligans* (95% MLBS and 1.00 BYPP; Fig. 1) in *Latorua*, concurring with previous studies (Crous et al. 2015a; Wang et al. 2023; Crous et al. 2024; Crous et al. 2025). The taxonomic revision of *Pseudoasteromassaria spadicea* and *Bahusandhika grootfonteinensis* is formally needed to be reclassified pending further study.

### Taxonomy

#### Polyschema adansoniae-digitatae

Taxon classificationFungiPleosporalesLatoruaceae

F.Q. Sun, Kumla, Phookamsak & Suwannar. sp. nov.

20DF5763-FDFF-5BCE-9491-A56F310B1B6E

MB861189

Fig. 2

##### Etymology.

The species epithet “adansoniae-digitatae” refers to the host, *Adansonia digitata*, from which the fungus was isolated.

**Figure 2.**
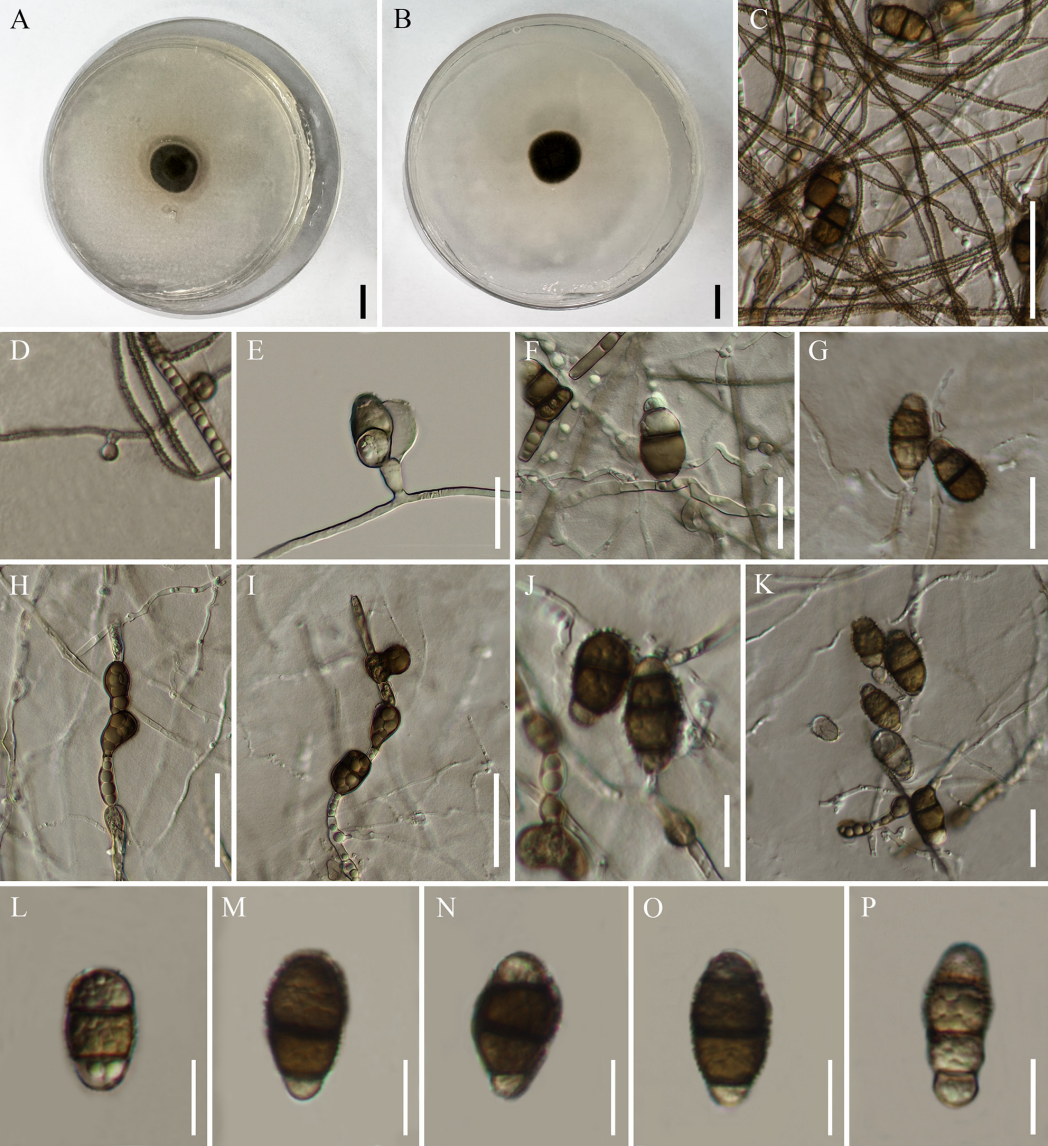
*Polyschema adansoniae-digitatae* (KUN-HKAS 150832, holotype). **A, B**. Colony on PDA from above and reverse; **C**. Hyphae; **D, E**. Conidiogenous cells with conidia; **F, G**. Conidia growing directly on hyphae; **H, I**. Chlamydospores; **J–P**. Conidia. Scale bars: 10 mm (**A, B**); 50 µm (**C**); 30 µm (**H, I, K**); 20 µm (**D–G**); 15 µm (**J**); 10 µm (**L–P**).

##### Type.

China • Yunnan Province: Honghe Prefecture, Honghe County, isolated from healthy root of baobab tree, 23°15'18"N, 102°14'12"E, elevation 459.8 m, 25 June 2024, F.Q. Sun, BR11-5, dried herbarium culture KUN-HKAS 150832 (**Holotype**, preserved in a metabolically inactive state), **ex-type living culture** KUNCC25-20156.

##### Description.

Dark-brown, filamentous, septate, endophyte isolated from healthy root of baobab tree (*Adansonia digitata*). ***Mycelia*** 1.7–2.2 µm wide, immersed to superficial, composed of branched, septate, initially hyaline to pale brown, smooth hyphae, later becoming brown to dark brown, finely verruculose or warty-surfaced hyphae. ***Conidiophores*** semi-macronematous or micronematous, hyaline, aseptate, or reduced to conidiogenous cells. ***Conidiogenous cells*** 4.0–6.9 × 2.8–5.6 µm (x̄ = 5.6 × 4.2 μm, *n* = 30), mono- or polytretic, solitary, discrete, globose or clavate, lateral, rarely terminal, pale brown, smooth-walled, with inconspicuous 1–2 conidiogenous loci. ***Conidia*** 18.5–27.6 × 9.7–14.3 µm (x̄ = 23.4 × 12.0 μm, *n* = 50), solitary to aggregated, acrogenous or catenate, in short branched chains, sometimes born directly on hyphae, ellipsoidal to obovoid, or oblong, pale brown, smooth-walled when young, becoming brown to dark brown at maturity, versicolored, paler brown at apical and basal cells, darker brown in the middle cells, (1–)2–3-septate, slightly constricted at septa, with thick and dark-brown, pigmented, echinulate or spikey warty surface; secondary conidia arising via apical cells. ***Chlamydospores*** 11.2–18.7 × 9.1–12.5 µm (x̄ = 15.6 × 11.0 μm, *n* = 50), terminal or intercalary, appear in short chains, varied in shape, usually globose to subglobose, or ovoid, dark brown, 0–1-septate, thick, smooth-walled.

##### Culture characteristics.

Colony grows slowly on PDA, reaching 12 mm in diameter after seven days at room temperature (25–30 °C) under dark conditions, floccose, raised, greyish green, white at margin when immature, becoming greyish green to almost blackish olive when mature, producing yellowish-brown pigment.

##### Additional material examined.

China • Yunnan Province: Honghe Prefecture, Honghe County, isolated from root of baobab tree, 23°15'18"N, 102°14'12"E, elevation 459.8 m, 25 June 2024, F.Q. Sun, BR11-5-1, **ex-paratype living culture**KUNCC 25-20157.

##### Notes.

The present phylogeny demonstrated that the new strains KUNCC 25-20156 (ex-holotype) and KUNCC 25-20157 (ex-paratype) are phylogenetically situated within the genus *Polyschema* (Fig. 1). The newly identified species, *P. adansoniae-digitatae*, has a close relationship with the clade including *P. congolense* and *P. hongheense*. Base pair comparisons in the ITS, LSU, and *rpb*2 loci between *P. adansoniae-digitatae* KUNCC25-20156 (holotype) and *P. congolense*CBS 542.73 (holotype) showed 10.1% nucleotide differences (58/575 bp, including 27 gaps) in ITS, 2.3% nucleotide differences (19/840 bp, including four gaps) in LSU, and 6.8% nucleotide differences (52/761 bp, no gaps) in *rpb*2. Due to the unavailability of the SSU sequence for *P. congolense*, comparative analyses for this locus could not be conducted. *Polyschema adansoniae-digitatae* KUNCC25-20156 and *P. hongheense*KUNCC 25-20158 showed nucleotide differences of 9.6% (52/544 bp, including 22 gaps) in ITS, 2.3% (19/834 bp, including four gaps) in LSU, 2.8% (38/1344 bp, including 18 gaps) in SSU, and 6.7% (70/1042 bp, including one gap) in *rpb*2, respectively.

Accompanied by phylogenetic evidence, morphological characteristics provide clear distinctions among *P. adansoniae-digitatae*, *P. congolense*, and *P. hongheense*, particularly in terms of conidial features. The conidia of *P. adansoniae-digitatae* are 2–3-septate, generally larger, and exhibit more variable shapes, ranging from ellipsoidal to obovoid or oblong, whereas those of *P. hongheense* are 1–2-septate (sometimes reaching up to four septa), more uniformly sized, and generally narrower (Table 2). Furthermore, conidiogenous cells of *P. adansoniae-digitatae* are significantly smaller than those of *P. congolense* and *P. hongheense* (Table 2).

**Table 2.** *Polyschema* species with a synopsis of the characteristics and relevant references.

Group	*Polyschema* species	Morphological characteristics				Sequence data	References
		Conidiophores	Conidiogenous cells	Conidia	Chlamydospores		
Group I	*P. adansoniae-digitatae*	Macronematous or micronematous, hyaline, aseptate, or reduced to conidiogenous cells	4–6.9 × 2.8–5.6 µm, globose or clavate, mono- or polytretic, discrete, smooth-walled	18.5–27.6 × 9.7–14.3 µm, (1–)2–3-septate, ellipsoidal to obovoid, or oblong, brown to dark brown, versicolored, echinulate	11.2–18.7 × 9.1–12.5 µm, terminal or intercalary, appear in short chains, globose to subglobose or ovoid, dark brown, 0–1-septate, thick, and smooth-walled	ITS, LSU, SSU, and *rpb*2	This study
	*P. chambalense*	Micronematous and finely echinulate	4–6 μm diam., spherical to subspherical, mono- or polytretic, discrete, sometimes in short chains	13.5–49.5 × 6–11.5 um, 3–9-septate, commonly with 4–6 septa, clavate, obclavate, cylindrical to sigmoid, pale brown, finely echinulate, mostly formed singly, sometimes in short chains	Absent	Unavailable	Joshi et al. (1983)
	*P. congolense*	Mostly reduced to conidiogenous cells	4.5–9.5 × 3–6 μm, spherical or enlarged at the apices, mono- or polytretic, discrete, smooth-walled, or slightly echinulate	16–26 × 8–11 μm, 2–3-septate, commonly with 2 septa, clavate, ellipsoidal, brown, finely echinulate	Absent	ITS, LSU, and *rpb*2	Reisinger and Kiffer (1974)
	*P. hongheense*	2.6–4.6 × 3.3–6.6 µm, short and subglobose, sometimes elongate, and curved, macronematous, or micronematous, or reduced to conidiogenous cells, septate, smooth to finely echinulate	4.6–6.9 × 4–8.9 µm, globose or clavate, mono- or polytretic, discrete, smooth-walled, or slightly echinulate	12.8–36.6 × 7.4–13.6 µm, 1–4-septate, commonly with 1–2 septa, fusoid-ellipsoidal, pale brown to dark brown, verruculose or echinulate	Absent	ITS, LSU, SSU, and *rpb*2	This study
	*P. indicum*	Absent	6–8 μm diam., spherical or subspherical, monotretic, discrete, verruculose or echinulate	15–30 × 10–14 μm, 2–4-septate, ellipsoidal, or pyriform, dark golden brown, echinulate or verruculose	Absent	Unavailable	Ellis (1976)
	*P. larviforme*	Semi-micromematous and verruculose	5–9 μm diam., spherical to subspherical, mono- or polytretic, discrete, solitary	30–80 × 16–20 μm, 4–12-septate, commonly with 7–10 septa, clavate, obclavate, or ellipsoidal, sometimes sigmoid, dark reddish brown, verrucose	Absent	ITS and LSU	Ellis (1976) and Joshi et al. (1983)
	*P. olivaceum*	Absent	6–9 μm diam., spherical or subspherical, monotretic, discrete, sometimes in short chains and flattened dorsiventrally, echinulate	17–37 × 6–9 μm, 2–7-septate, cylindrical, pale to mid golden brown, verruculose or echinulate	Absent	Unavailable	Ellis (1976)
	*P. queenslandicum*	Absent	6–10 × 5–6.5 μm, subglobose or clavate, mono- or polytretic, discrete, verruculose	20–36 × 9–13 µm, 2–4-septate, commonly with 3 septa, cylindrical to ellipsoidal, pale brown at both ends, dark brown at the middle, verruculose	Absent	Unavailable	Matsushima (1989)
	*P. radicicola*	7.2–10.3 × 3.4–6.1 µm, unbranched, clavate or short cylindrical, micronematous, or reduced to conidiogenous cells	4.4–7.2 × 4.3–7.3 µm, globose, clavate or short cylindrical, monotretic, discrete, smooth, or slightly verrucose	20.4–40.9 × 7.5–13.1 µm, 2–5-septate, cylindrical, pale brown, verruculose or echinulate	Absent	ITS, LSU, SSU, and *rpb*2	This study
	*P. sagari*	6.6–59.4 × 3.3–4.9 μm, unbranched or frequently bifurcated at base, 1–4-septate, smooth, or rarely echinulate	6.6–13.2 × 3.3–4.9 μm, globose, mono- or polytretic, discrete, smooth, or finely echinulate	5–20 × 3–8 µm, 1–5-septate, commonly with 3 septa, cylindrical, pale brown, echinulate	Absent	Unavailable	Pandeya et al. (1978)
Group I	*P. sclerotigenum*	Up to 45 µm long, 2.5–3 µm wide, unbranched, subcylindrical, smooth or verruculose	5–7.5 × 5–7 µm, spherical to subspherical, monotretic, rarely polytretic, discrete, sometimes in short chains, verruculose to tuberculate	36–65(–75) × 7–8 µm, 5–16-septate, cylindrical, or sigmoid, brown to dark brown, verruculose to tuberculate, with *Sclerotium*-like structures often present	Absent	ITS and LSU	Crous et al. (2015b)
	*P. terricola*	Absent	4–8 μm diam., spherical to subspherical, mostly monotretic, discrete, smooth or verruculose	15–35 × 13–18 μm, 0–3-septate transversally, and sometimes 1 longitudinal or oblique septum, subspherical, pyriform or clavate, dark brown, verruculose	8–15 μm diam.	ITS, LSU, SSU, and *rpb*2	Ellis (1976)
	*P. toruloides*	6–9(–16) μm long, clavate or doliiform, 0–1-septate, slightly verruculose	6.5–8.5 × 6–8 μm, globose or widely obovate, mostly polytretic, discrete, verruculose	(15–)26–73 × 7–10 μm, (2–)3–11-septate, cylindrical, brown, finely verruculose	Absent	Unavailable	Matsushima (1980)
	*P. variabile*	Semi-micronematous and verruculose	4.5–5.5 × 3–4.5 μm, subglobose (or 9.5 × 4 μm, clavate), mono- or polytretic, discrete, sometimes in short chains	9.5–28.5 × 6–11.5 μm, 0–5-septate, commonly with 2–3 septa, clavate, straight or curved, rarely sigmoid, greyish brown to dark brown, finely echinulate, mostly solitary or in pairs, sometimes in short chains of 2–3	12.5–17.5 × 10.5–14.5 μm, numerous, solitary in chains or in dense clusters, globose to oval, occasionally with transverse and longitudinal septa, verrucose or smooth	Unavailable	Tiwari et al. (1977) and Joshi et al. (1983)
	*P. yakuense*	Micronematous	Intercalary, incorporated in repent and aerial hyphae, laterally forming one or more conidia directly	(13–)15–22 × 6–9 μm, (2–)3-septate, cylindrical to elliptical, dark brown at the middle, subhyaline at both ends, smooth to verruculose	Absent	Unavailable	Matsushima (1975)
Group II	*P. amoenum*	25–50 × 3–4 µm, mostly moniliform, semi-macronematous, mononematous, smooth-walled	5–8 × 3–3.5 µm, globose, doliiform to ellipsoid, monotretic, determinate, terminal, sometimes sympodially proliferation become intercalary, smooth-walled	25–29 × 8–10 µm, 4–5-septate, mostly fusiform to navicular, rarely clavate, dark brown to black at the middle, pale brown at both ends, smooth-walled, with a schizolytic conidial secession	Absent	Unavailable	Castañeda Ruíz et al. (2009)
	*P. bicellulare*	Absent	3–5 µm diam., spherical to subspherical, monotretic, discrete, smooth-walled	10–12 × 5–6 µm, 1-septate, cylindrical to clavate, reddish brown, smooth-walled, with germ pore present	Absent	Unavailable	Shearer (1982)
	*P. clavulatum*	Micronematous, mononematous, integrated, inconspicuous	Smooth, 4–7 μm in diam., spherical or subspherical, monotretic, smooth-walled	30–46 × 12–16 μm, 3–5-septate, cylindrical to clavate, pale to rather dark reddish brown with a paler basal cell, smooth-walled	Absent	Unavailable	Ellis (1976) and Delgado-Zúñiga et al. (2024)
	*P. cubense*	Micronematic or semi-macronematic	4–8.5 × 4.6 μm, subspherical or ampulliform, monotretic, smooth-walled	11–20 × 4.8–8 μm, 1–3-septate, cylindrical to ellipsoidal or clavate, brown to dark brown, smooth-walled	Absent	Unavailable	Mercado-Sierra and Mena-Portales (1992)
	*P. endophytica*	Up to 45 μm tall, 3–5 μm wide, micronematous, mononematous	6–8.5 × 2–7 μm, hemispherical, subspherical or cylindrical, monotretic, discrete, smooth-walled	Aseptate, subglobose to ellipsoidal, dark brown, smooth-walled	Absent	ITS and LSU	Crous et al. (2025)
	*P. lignicola*	Micronematous, mononematous	2.8–4.2 μm diam., spherical or subspherical, monotretic, rarely polytretic, discrete, or integrated, determinate, sometimes in short chains of 2–3, smooth-walled or verrucose	14–21 μm long, 4.2–7 μm at base, and 7–11.2 μm wide at apex, always 1-septate, obovoid or pyriform, pale to mid dark brown, smooth-walled	Formed in culture	Unavailable	Rao and Reddy (1984)
Group II	*P. nigroseptatum*	20–38 × 5–7 µm, moniliform, smooth-walled	8–10 × 7–10 µm, globose or clavate, monotretic, determinate, smooth-walled	41–45 × 19–22 µm, 2–3(–5)-septate, clavate to ovoid, brown, dark brown to black at the septa, smooth-walled	Absent	Unavailable	Mota et al. (2008)
	*P. obclaviforme*	11–20 × 4–5 µm, moniliform, smooth-walled	5–6 × 3–5 μm, globose or clavate, monotretic, determinate, smooth-walled	30–48 × 6–7 μm, (4–)5-septate, obclavate, brown at the middle and pale brown at the ends, smooth-walled, seceding schizolytically	Absent	Unavailable	Ruiz et al. (2000)
	*P. ylnenei*	Micronematous, mononematous	3–4.5 μm diam., spherical, monotretic, determinate, smooth-walled	21–31 × 7–11 μm, usually 3–5-septate transversally, and generally one vertical septum towards the lower end, mid brown to dark or blackish brown, dark brown to black at the septa, smooth-walled	Absent	Unavailable	Manoharachary et al. (2006)

#### Polyschema hongheense

Taxon classificationFungiPleosporalesLatoruaceae

F.Q. Sun, Kumla, Phookamsak & Suwannar. sp. nov.

F3CCFE63-F074-57B3-9B17-4E6FC4F3B35D

MB861247

Fig. 3

##### Etymology.

The species epithet “hongheense” refers to the locality, Honghe County, Yunnan, China, from where the fungus was collected.

**Figure 3.**
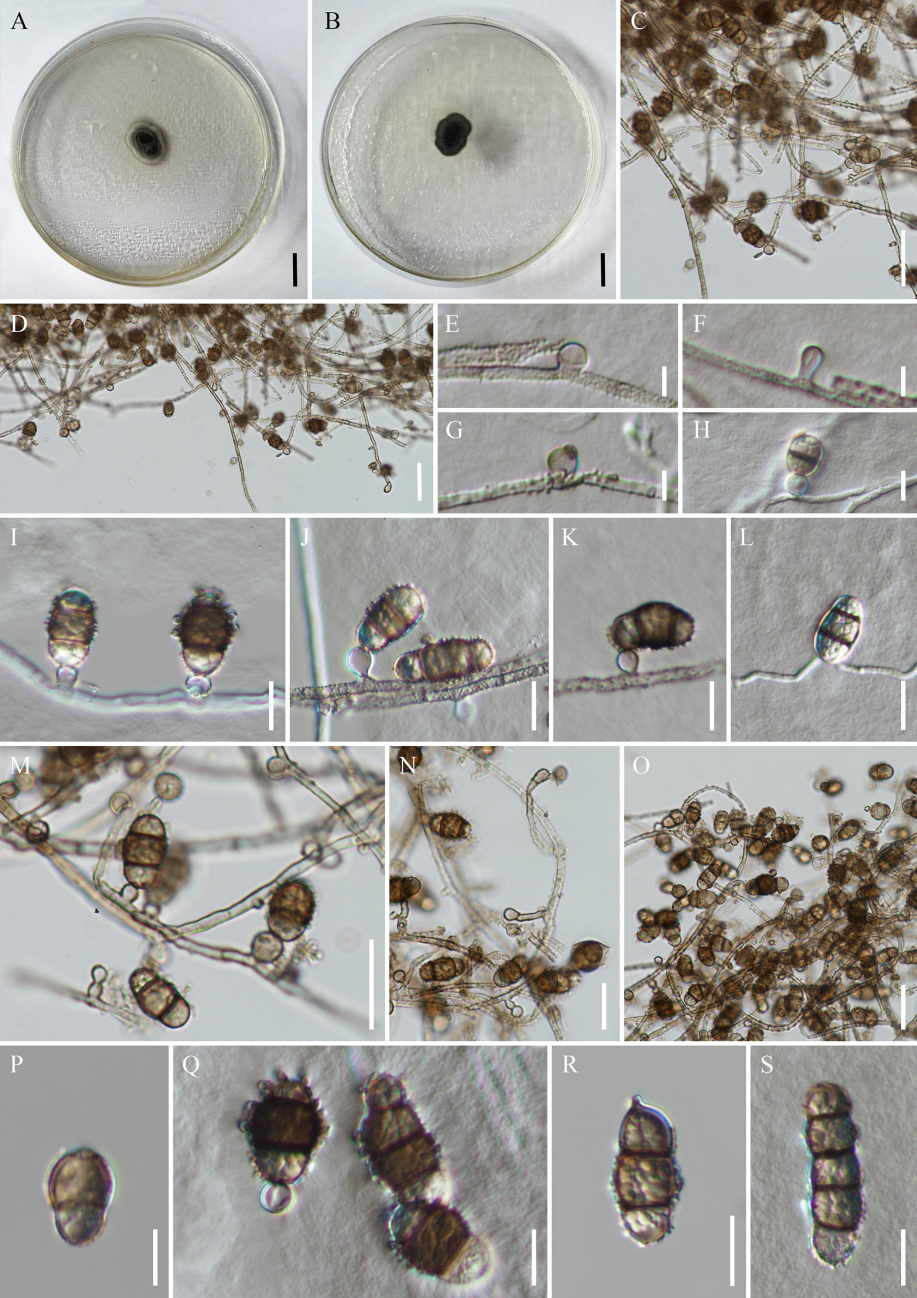
*Polyschema hongheense* (KUN-HKAS 150833, holotype). **A, B**. Culture from above and reverse; **C, D**. Hyphae; **E–H**. Conidiogenous cells; **I**. Conidia borne parallel to the axis of the conidiogenous cell; **J**. Conidia borne obliquely to the axis of the conidiogenous cell; **K**. Conidia borne on the axis of the conidiogenous cell but obliquely oriented; **L**. Conidia growing directly on hyphae; **M, N**. Conidiophores; **O**. Conidia in mass; **P–S**. Conidia. Scale bars: 10 mm (**A, B**); 20 µm (**C, D, M–O**); 10 µm (**I–L, P–S**); 5 µm (**E–H**).

##### Type.

China • Yunnan Province: Honghe Prefecture, Honghe County, isolated from healthy root of baobab tree, 23°15'18"N, 102°14'12"E, elevation 459.8 m, 25 June 2024, F.Q. Sun, BR11-10, dried herbarium culture KUN-HKAS 150833 (**Holotype**, preserved in a metabolically inactive state), **ex-holotype living culture** KUNCC25-20158.

##### Description.

Dark brown, filamentous, septate, endophyte isolated from healthy root of baobab tree (*Adansonia digitata*). ***Mycelia*** 2.2–4.3 µm wide, superficial, composed of branched, narrow, septate, hyaline to light brown hyphae, smooth-walled when young, coarsely roughened, minutely echinulate or verruculose when mature. ***Conidiophores*** 2.6–4.6 × 3.3–6.6 µm (x̄ = 3.7 × 5.0 μm, *n* = 50), macronematous or micronematous, solitary, erect or flexuous, hyaline to pale brown, unbranched, usually short and subglobose, sometimes elongated and curved, septate, 2.4–2.5 × 11.2–38.5 µm, or reduced to conidiogenous cells. ***Conidiogenous cells*** 4.6–6.9 × 4.0–8.9 µm (x̄ = 5.8 × 6.5 μm, *n* = 30), mono- or polytretic, solitary, globose or clavate, hyaline, smooth-walled. ***Conidia*** solitary, borne parallelly or obliquely to main axis of the conidiogenous cells, sometimes borne on the axis of conidiogenous cells, but obliquely oriented, sometimes arising directly from hyphae, fusoid-ellipsoidal, slightly constricted at septa, hyaline when young, brown when mature, paler at end cells, dark brown to black at the centre, verrucose to echinulate at surface, (1–4)-septate, usually (1–2)-septate; 1-septate measuring 12.8–19.8 × 7.4–12.7 µm (x̄ = 16.5 × 10.2 μm, *n* = 50), 2-septate 16.5–22.4 × 9.5–13.6 µm (x̄ = 19.6 × 11.6 μm, *n* = 50), 3-septate 20.0–32.7 × 8.0–11.8 µm (x̄ = 26.5 × 10.0 μm, *n* = 50), 4-septate 34.5–36.6 × 10.1–12.6 µm (x̄ = 35.6 × 11.4 μm, *n* = 50).

##### Culture characteristics.

Colony grows slowly on PDA, reaching 14 mm in seven days at room temperature (25–30 °C) under dark conditions, colonies irregular, fluffy asteroid, raised, brown, white at the margin when immature, becoming completely brown to olive brown when mature.

##### Notes.

*Polyschema hongheense* (KUNCC25-20158) formed a distinct lineage sister to *P. congolense*, supported by significant statistical values (99% MLBS and 1.00 BYPP; Fig. 1), which indicates a close phylogenetic relationship. Base pair comparisons in the ITS, LSU, and *rpb*2 loci between *P. hongheense* KUNCC25-20158 (holotype) and *P. congolense*CBS 542.73 (holotype) showed 3.3% nucleotide differences (17/521 bp, including five gaps) in ITS, 0.7% nucleotide differences (6/845 bp, no gaps) in LSU, and 5.9% nucleotide differences (45/760 bp, including one gap) in *rpb*2. Due to the absence of SSU sequences for *P. congolense*, comparisons at this locus were not available.

Morphologically, *P. hongheense* can be distinguished from *P. congolense* by its wider mycelium (2.2–4.3 µm wide vs. 1.5–2.5 μm wide) and conidia that are larger (12.8–36.6 × 7.4–13.6 µm vs. 16–26 × 8–11 μm) and reach up to four septa, whereas *P. congolense* has 2–3-septate, commonly with two septa (Table 2).

#### Polyschema radicicola

Taxon classificationFungiPleosporalesLatoruaceae

F.Q. Sun, Kumla, Phookamsak & Suwannar. sp. nov.

942E2874-E09E-5BD3-90A2-297CD53CEC5B

MB861248

Fig. 4

##### Etymology.

The species epithet “radicicola” refers to the root from which the species was isolated.

**Figure 4.**
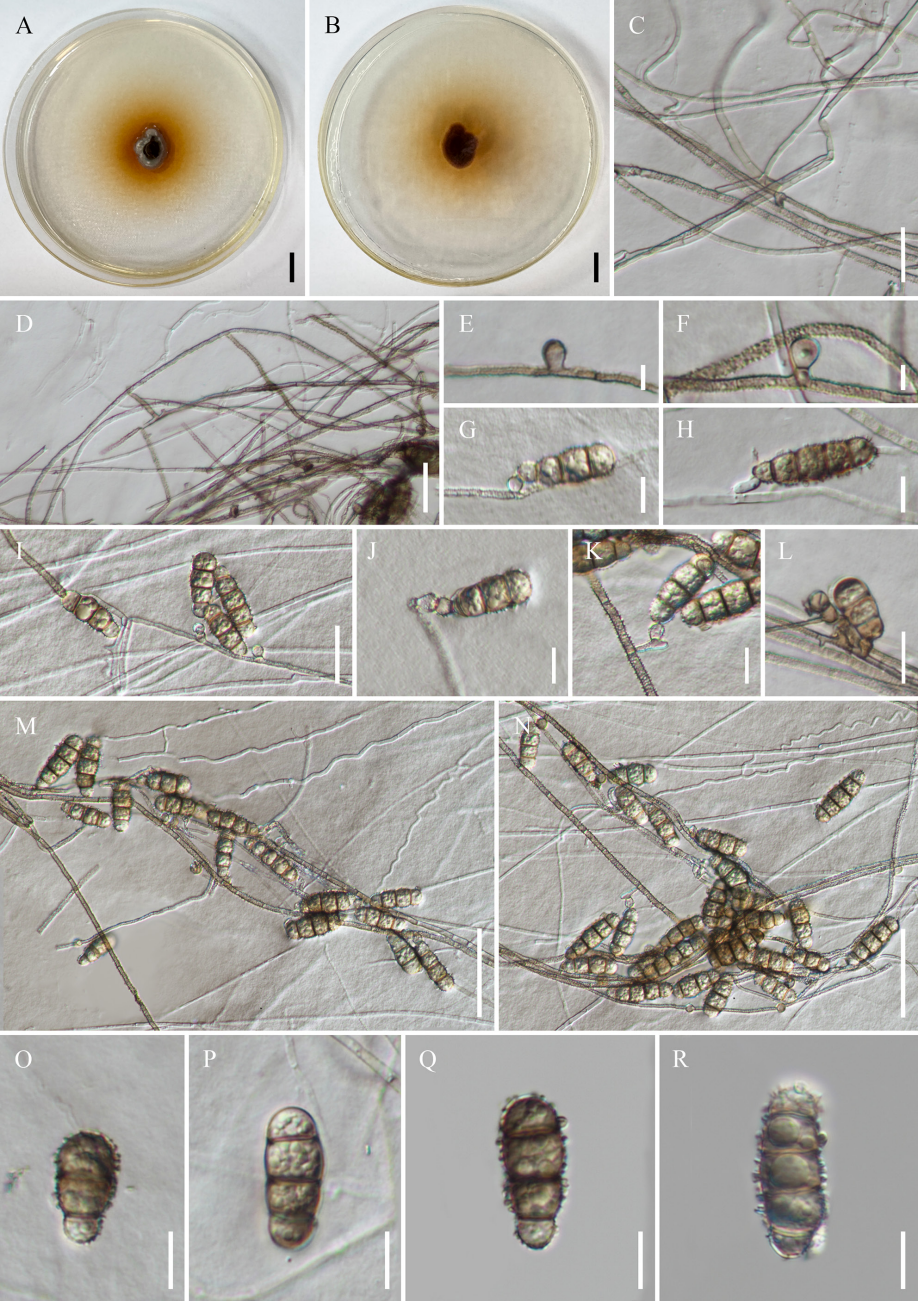
*Polyschema radicicola* (KUN-HKAS 150834, holotype). **A, B**. Culture from above and reverse; **C, D**. Hyphae; **E–H**. Conidiogenous cells; **I**. Conidia growing terminally; **J, K**. Conidiophores; **L–N**. Conidia in mass; **O–R**. Conidia. Scale bars: 10 mm (**A, B**); 50 µm (**M, N**); 20 µm (**C**); 15 µm (**L**); 10 µm (**D, G–K, O–R**); 5 µm (**E, F**).

##### Type.

China • Yunnan Province: Honghe Prefecture, Honghe County, isolated from healthy root of baobab tree, 23°25'21"N, 102°14'19"E, elevation 503.7 m, 25 June 2024, F.Q. Sun, BR16-1, dried herbarium culture KUN-HKAS 150834 (**Holotype**, preserved in a metabolically inactive state), **ex-type living culture** KUNCC25-20159.

##### Description.

Dark brown, filamentous, septate endophyte (DSE) isolated from healthy root of baobab tree (*Adansonia digitata*). ***Mycelium*** 1.7–4.0 µm wide, superficial, composed of branched, narrow, septate, hyaline to light brown hyphae, flexuous hyphae, smooth-walled when young, straight or curved, coarsely roughened when mature, minutely echinulate or verruculose. ***Conidiophores*** 7.2–10.3 × 3.4–6.1 µm (x̄ = 8.8 × 4.8 μm, *n* = 50), micronematous, solitary, erect, hyaline to pale brown, unbranched, clavate, or short cylindrical, or reduced to conidiogenous cells. ***Conidiogenous cells*** 4.4–7.2 × 4.3–7.3 µm (x̄ = 5.8 × 5.9 μm, *n* = 30), monotretic, solitary, terminal or lateral, globose, clavate or short cylindrical, hyaline, smooth-walled. ***Conidia*** solitary, mostly pleurogenous, at times acrogenous, usually borne along the axis of conidiogenous cells, but sometimes obliquely oriented, cylindrical, slightly constricted at septa, pale brown, end cells paler, dark brown to black at the septa, verrucose to echinulate surfaced, (2–5)-septate, 20.4–40.9 × 7.5–13.1 µm (x̄ = 31.0 × 10.5 μm, *n* = 50).

##### Culture characteristics.

Colony grows slowly on PDA, reaching 15 mm in seven days at room temperature (25–30 °C) under dark conditions, colonies irregular, felted, slightly heaped and folded, pale brown when immature, becoming completely brown when mature, producing orange pigment.

##### Additional materials examined.

China • Yunnan Province: Honghe Prefecture, Honghe County, isolated from healthy root of baobab tree, 23°25'21"N, 102°14'19"E, elevation 503.7 m, 25 June 2024, F.Q. Sun, BR16-5, **ex-paratype living culture**KUNCC 25-20160.

##### Notes.

*Polyschema radicicola* is basal to a subclade containing *P. adansoniae-digitatae* (KUNCC25-20156 and KUNCC25-20157), *P. congolense* (CBS 542.73), and *P. hongheense* (KUNCC 25-20158). These species form a subclade and have a close relationship with *P. endophytica* (COAD3972) and *P. terricola* (CBS 301.65). Based on nucleotide comparisons, *P. radicicola* KUNCC25-20159 (holotype) differs from *P. congolense*CBS 542.73 (holotype) in the ITS sequence by 34/548 bp (6.2%, including 14 gaps), in the LSU sequence by 14/846 bp (1.7%, including one gap), and in the *rpb*2 sequence by 82/768 bp (10.7%, including three gaps). *Polyschema radicicola* KUNCC25-20159 differs from *P. hongheense* KUNCC25-20158 by 5.8% nucleotides in ITS (30/518 bp, including nine gaps), 1.4% nucleotides in LSU (12/844 bp, including one gap), 2.3% nucleotides in SSU (31/1336 bp, including nine gaps), and 8.9% nucleotides in *rpb*2 (94/1060 bp, including four gaps). *Polyschema radicicola* KUNCC25-20159 differs from *P. endophytica* COAD3972 (holotype) by 9.4% nucleotides in ITS (52/556 bp, including 28 gaps) and 2.0% nucleotides in LSU (17/849 bp, including four gaps), and differs from *P. terricola*CBS 301.65 (holotype) by 11.4% nucleotides in ITS (57/501 bp, including 33 gaps), 2.3% nucleotides in LSU (20/852 bp, including seven gaps), and 10.0% nucleotides in *rpb*2 (77/769 bp, including three gaps).

Morphologically, *Polyschema radicicola* is distinguishable by its colony producing orange pigment (Table 2). Furthermore, *P. radicicola* exhibits mycelial hyphae that are occasionally narrow, flexuous, and smooth-walled, yet remain capable of generating conidiogenous cells and conidia. Conidia of *P. radicicola* differ from those of *P. terricola* (Ellis 1976) by being longer and narrower (20.4–40.9 × 7.5–13.1 µm vs. 15–35 × 13–18 μm), cylindrical, distinctly echinulate at the surface, and transversely 2–5-septate, lacking vertical septa, whereas *P. terricola* has transversely 0–3-septate conidia with sometimes one longitudinal or oblique septum (Ellis 1976). *Polyschema congolense* (Reisinger and Kiffer 1974) can be distinguished from *P. radicicola* by having smaller (16–26 × 8–11 μm) and 2–3-septate conidia, commonly with two septa. *Polyschema hongheense* differs by having shorter (12.8–36.6 × 7.4–13.6 µm), paler brown, 1–4-septate conidia, commonly with 1–2 septa and occasionally formed terminally. *Polyschema endophytica* (Crous et al. 2025) differs by having aseptate, globose, subglobose to ellipsoidal, smooth-walled conidia.

## Discussion

Species of *Polyschema* were divided into two groups by Rao and Reddy (1984) based on their conidial surfaces and were further divided by conidial septa, shapes, colors, and sizes. This grouping was later updated by Mota et al. (2008). Group I has conidia with ornamented walls, whereas Group II has smooth-walled conidia. Based on their conidial surfaces, the three new *Polyschema* species, *P. adansoniae-digitatae*, *P. hongheense*, and *P. radicicola*, obtained in this study were classified into Group I (ornamented-walled conidia). A morphological comparison between our new species and other *Polyschema* species is provided in Table 2. However, morphological classification in *Polyschema* remains limited by ambiguous descriptions; therefore, integrating morphological data with molecular phylogenetic analysis provides a more robust and reliable framework for identifying and confirming new species. By adding our newly introduced species, there are a total of 24 species accommodated in *Polyschema* (Tables 2, 3). However, only eight species are confirmed with molecular evidence (Table 2). Furthermore, most sequences in these eight species belong to ITS and LSU; therefore, previous phylogenies including *Polyschema* (Crous et al. 2024; Crous et al. 2025) relied mainly on ribosomal DNA regions (ITS and LSU). The present study employs a combined analysis of ITS, LSU, SSU, and *rpb*2 regions, which is mostly consistent with earlier approaches but provides clearer phylogenetic relationships among closely related species with stronger statistical support. The phylogenetic reconstruction reveals a progressive, stepwise pattern of separation within species of *Polyschema*. Through nucleotide comparisons in the present study while introducing *P. adansoniae-digitatae*, *P. hongheense*, and *P. radicicola*, these comparisons indicate minor differences in LSU and SSU loci, while there are significant base pair differences in ITS and *rpb*2. Therefore, it suggests that ITS and *rpb*2 sequences are efficient for resolving the ambiguous placement of *Polyschema* species in the future.

**Table 3.** Updated genera and species in Latoruaceae with their life mode, habitat, isolation sources, and geographic contributions.

Genera	Species	Life mode	Habitat	Isolation source	Geographic contribution	References
* Latorua *	*L. caligans*	Saprobic	Terrestrial	Soil	Brazil	Crous et al. (2015a)
	*L. grootfonteinensis*	Saprobic	Terrestrial	Brown sandy soil	South Africa	Crous et al. (2015a)
* Matsushimamyces *	*M. bohaniensis*	Saprobic	Terrestrial	Soil	India	Sharma et al. (2015)
	*M. venustus*	Saprobic	Terrestrial	Decaying leaf	Cuba	Ruiz et al. (1996)
* Multiverruca *	*Mu. sinensis*	Saprobic	Terrestrial	Soil	China (Zhejiang)	Wang et al. (2023)
* Polyschema *	*P. adansoniae-digitatae*	Endophytic	Terrestrial	Baobab roots	China (Yunnan)	In this study
	*P. amoenum*	Saprobic	Terrestrial	Twig of unidentified plant	Venezuela	Castañeda Ruíz et al. (2009)
	*P. bicellulare*	Saprobic	Terrestrial	Twig of *Platanus occidentalis*	USA (Illinois)	Shearer (1982)
	*P. chambalense*	Saprobic	Terrestrial	Soil	India	Joshi et al. (1983)
	*P. clavulatum*	Saprobic	Terrestrial	Fallen wood of *Quercus laurina* and *Q. castanea*, decorticated wood of *Eucalyptus*, and mixed oak forest	Mexico and USA (California)	Ellis (1976) and Delgado-Zúñiga et al. (2024)
	*P. congolense*	Saprobic	Terrestrial	Tropical forest soil	Democratic Republic of the Congo	Reisinger and Kiffer (1974)
	*P. cubense*	Saprobic	Terrestrial	Dead leaves and petioles of *Roystonea regia*	Cuba	Mercado-Sierra and Mena-Portales (1992)
	*P. endophytica*	Endophytic	Terrestrial	Healthy roots of coffee plant	Brazil	Crous et al. (2025)
	*P. hongheense*	Endophytic	Terrestrial	Baobab roots	China (Yunnan)	In this study
	*P. indicum*	Saprobic	Terrestrial	Grassland soil	India	Ellis (1976)
	*P. larviforme*	Saprobic	Terrestrial	The cut surface of old firewood	USA (New York)	Ellis (1976)
	*P. lignicola*	Saprobic	Terrestrial	The decorticated wood	India	Rao and Reddy (1984)
	*P. nigroseptatum*	Saprobic	Terrestrial	Mangrove debris	Mexico	Mota et al. (2008)
	*P. obclaviforme*	Saprobic	Terrestrial	Fallen, decaying leaves of an unidentified plant in rainforest	Cuba	Ruiz et al. (2000)
	*P. olivaceum*	Saprobic	Terrestrial	Old culms of *Zea mays* and isolated from the air	USA (New Jersey)	Ellis (1976)
	*P. queenslandicum*	Saprobic	Terrestrial	Forest soil	Australia	Matsushima (1989)
	*P. radicicola*	Endophytic	Terrestrial	Baobab roots	China (Yunnan)	In this study
	*P. sagari*	Saprobic	Terrestrial	Red-colored soil of *Patharia* forest	India	Pandeya et al. (1978)
	*P. sclerotigenum*	Pathogenic	Terrestrial	Human clinic tissue	USA (Texas)	Crous et al. (2015b)
	*P. terricola*	Saprobic	Terrestrial	Soil	Brazil	Ellis (1976)
	*P. toruloides*	Saprobic	Terrestrial	Leaves of *Acacia confusae*	China (Taiwan)	Matsushima (1980)
	*P. variabile*	Saprobic	Terrestrial	Soil	India	Tiwari et al. (1977)
	*P. yakuense*	Saprobic	Terrestrial	Forest soil	Japan	Matsushima (1975)
	*P. ylnenei*	Saprobic	Terrestrial	Dead unidentified twigs	India	Manoharachary et al. (2006)
* Pseudoasteromassaria *	*Ps. aquatica*	Saprobic	Freshwater	Decaying wood submerged in freshwater	Thailand	Dong et al. (2020)
	*Ps. fagi*	Pathogenic	Terrestrial	Twigs of *Fagus crenata*	Japan	Ariyawansa et al. (2015)
	*Ps. spadicea*	Saprobic	Freshwater	Submerged wood in freshwater	Thailand	Tibpromma et al. (2017)
* Triseptata *	*T. sexualis*	Saprobic	Terrestrial	Dried branches of an unidentified plant	Thailand	Boonmee et al. (2020)
	*T. podargi-strigoidis*	Saprobic	Terrestrial	Decayed wood of *Acacia* sp.	Australia	Tan and Bishop-Hurley (2024)
* Verrucohypha *	*V. endophytica*	Endophytic	Terrestrial	Macaw palm roots	Brazil	Crous et al. (2024)

Members of the family Latoruaceae play various ecological roles, including endophytic, pathogenic, and saprobic life modes (Table 3). Most *Polyschema* species have been documented as saprobes on various hosts, including soil, decaying wood, and leaves worldwide (Ariyawansa et al. 2015; Crous et al. 2015b; Wang et al. 2023; Table 3). More recent findings, including *P. sclerotigenum* from human clinical material (Crous et al. 2015b) and *P. endophytica* (Crous et al. 2025) as a root endophyte of coffee, suggest that the genus is not limited to a saprobic lifestyle. The three root endophytes introduced in this study, representing the first records of *Polyschema* from mainland China, further underscore its ecological plasticity. Furthermore, this is the first report of members of *Polyschema* living in baobab trees. Other genera within the Latoruaceae are mostly isolated from soil and decaying or decayed wood. The identification of *Pseudoasteromassaria aquatica* (Dong et al. 2020) and *Ps. spadicea* (Tibpromma et al. 2017) expanded the known habitats of this family to freshwater environments. *Pseudoasteromassaria fagi* (Ariyawansa et al. 2015) was reported as pathogenic on decaying wood submerged in freshwater in Thailand, exhibiting a lifestyle similar to *Polyschema sclerotigenum*. *Verrucohypha endophytica* (Crous et al. 2024) was discovered as an endophyte, a lifestyle also observed in *Polyschema endophytica* (Crous et al. 2025) and in the three new taxa introduced in this study. These observations point to a family characterized by ecological plasticity, morphological variability, and an expanding distribution range. Continued sampling from varied habitats will be crucial to uncover hidden knowledge of *Polyschema*, species diversity in Latoruaceae, and their extended ecological significance.

There is a controversial point in the present phylogeny. *Bahusandhika grootfonteinensis* is clustered with *Latorua* and remained distinct from the *Bahusandhika* group within Lentimurisporaceae, concurring with previous studies (Crous et al. 2015a; Wang et al. 2023; Crous et al. 2024; Crous et al. 2025). *Latorua grootfonteinensis* was transferred to *Bahusandhika* by Crane and Miller (2016) due to its similarities to *Bahusandhika* in conidiogenous cells, conidial development, overall conidial morphology, and lack of molecular data support. However, the present phylogeny and the phylogenies in Wang et al. (2023) and Crous et al. (2024; Crous et al. 2025) do not support this synonym. Therefore, *B. grootfonteinensis* should be reinstated in *Latorua* as *L. grootfonteinensis*. Similarly, *Pseudoasteromassaria spadicea* (MFLUCC 15-0972) clustered within *Triseptata* and remained distant from *Pseudoasteromassaria*. This species should be reclassified within *Triseptata* based on phylogenetic analyses. However, further work is needed to confirm the taxonomic placement of these two species, particularly by using more taxa sampling and morphological determination.

## Supplementary Material

XML Treatment for Polyschema adansoniae-digitatae

XML Treatment for Polyschema hongheense

XML Treatment for Polyschema radicicola

## Additional information

### Use of AI

No use of AI was reported.

### Author ORCIDs

Fangqi Sun https://orcid.org/0009-0009-7859-5474

Hongbo Jiang https://orcid.org/0000-0001-9655-4662

Jaturong Kumla https://orcid.org/0000-0002-3673-6541

Rungtiwa Phookamsak https://orcid.org/0000-0002-6321-8416

Junfu Li https://orcid.org/0009-0008-6088-2072

Jianchu Xu https://orcid.org/0000-0002-2485-2254

Jayarama Darbhe Bhat https://orcid.org/0000-0002-3800-5910

Nakarin Suwannarach https://orcid.org/0000-0002-2653-1913

### Data availability

All of the data that support the findings of this study are available in the main text.
